# RETRACTION: Bees' Honey Attenuation of Metanil-Yellow-Induced Hepatotoxicity in Rats

**DOI:** 10.1155/ecam/9820146

**Published:** 2025-11-06

RETRACTION: A. L. Al-Malki and A. A. Radwan Sayed, “Bees' Honey Attenuation of Metanil-Yellow-Induced Hepatotoxicity in Rats,” *Evidence-Based Complementary and Alternative Medicine* 2013 (2013), https://doi.org/10.1155/2013/614580.

The above article, published online on 3 June 2013 in Wiley Online Library (https://wileyonlinelibrary.com), has been retracted by John Wiley & Sons Ltd.

The retraction has been agreed following an investigation of the concerns raised by *Actinopolyspora biskrensis* on PubPeer [1]. More specifically, the tissue shown in Figure 5(g) appears identical to an image in Figure 2(b) of [2], where it is described as tissue of a different animal species subjected to different experimental conditions.

As a result of this investigation, the results of the article are no longer considered reliable.

The authors were informed of this retraction but did not provide a response.
